# Associations of Vitamin D With GPX4 and Iron Parameters in Chronic Obstructive Pulmonary Disease Patients: A Case–Control Study

**DOI:** 10.1155/2024/4505905

**Published:** 2024-10-28

**Authors:** Jun Fei, Ling Liu, Jia-Fei Li, Qiang Zhou, Yu Wei, Ting-Dong Zhou, Lin Fu

**Affiliations:** ^1^Department of Respiratory and Critical Care Medicine, Second Affiliated Hospital of Anhui Medical University, Hefei 230601, Anhui, China; ^2^Institute of Respiratory Diseases, Second Affiliated Hospital of Anhui Medical University, Hefei 230601, Anhui, China; ^3^Anhui Province Key Laboratory of Clinical and Preclinical Research in Respiratory Disease, Department of Pulmonary and Critical Care Medicine, First Affiliated Hospital, Bengbu Medical College, Bengbu 233004, Anhui, China; ^4^Department of Respiratory and Critical Care Medicine, People's Hospital of Yingshan, Fuyang 236000, Anhui, China; ^5^Department of Respiratory and Critical Care Medicine, First People's Hospital of Chuzhou, Chuzhou 239001, Anhui, China; ^6^Department of Clinical Laboratory, Second Affiliated Hospital of Anhui Medical University, Hefei, Anhui 230601, China; ^7^Center for Big Data and Population Health of IHM, Anhui Medical University, Hefei, Anhui 230032, China

**Keywords:** 25-hydroxyvitamin D, COPD patients, epidemiology, ferritin, glutathione peroxidase 4

## Abstract

**Background:** Vitamin D deficiency elevates the risk of chronic obstructive pulmonary disease (COPD) patients. Iron parameters elevation and glutathione peroxidase 4 (GPX4) reduction are involved in the process of COPD. The goal is to explore the associations of vitamin D with GPX4 and iron parameters in COPD patients through a case–control study.

**Methods:** COPD patients and control subjects were enrolled. Serum samples and lung tissues were collected. Serum vitamin D and iron levels and pulmonary ferritin and GPX4 expressions were determined. In addition, human pulmonary epithelial cells (BEAS-2B) were incubated with 1,25(OH)_2_D_3_ (100 nM), the active form of vitamin D3. Then, vitamin D receptor (VDR) and nuclear factor (erythroid-derived 2)-like 2 (Nrf-2) signaling were detected.

**Results:** In patients with COPD, serum 25-hydroxyvitamin D (25(OH)D) decreased, and iron and ferritin levels in serum and lung tissues increased. Furthermore, pulmonary expression of GPX4 was reduced. Correlative analyzes indicated that lung function was inversely correlated with iron parameters and positively correlated with GPX4. The results showed that serum 25(OH)D deficiency was associated with an elevation in serum iron parameters and a reduction in pulmonary GPX4. In addition, VDR- and Nrf-2-positive lung nuclei were decreased in COPD patients than in control subjects. In patients with COPD, the results indicated a positive relationship between VDR and Nrf-2. Further analysis revealed that Nrf-2-positive nuclei were negatively correlated with iron parameters. *In vitro* experiments found that 1,25(OH)_2_D_3_ treatment activated VDR signaling and elevated the expression of Nrf-2 and GPX4 in BEAS-2B cells.

**Conclusions:** Vitamin D deficiency is positively associated with GPX4 reduction and iron parameters elevation in COPD patients. It is recommended to explore the role of vitamin D supplementation in the progression of COPD.

## 1. Introduction

Chronic obstructive pulmonary disease (COPD) is characterized by persistent expiratory airflow limitation which is not completely reversible, excessive chronic inflammation, and destruction of small airways [1–3]. Recent studies have indicated that the number of COPD is estimated to be 3 million [4]. Due to the high mortality rate and enormous medical expense, COPD has become a vital public health challenge [5, 6]. Cigarette smoke, biomass fuels, air pollution, heavy metals, gases, dust, and fumes are the known risk factors for COPD [7–9]. Vitamin D and vitamin D receptor (VDR) draw more and more attention as vitamin D exerts an indispensable role in the uptake of calcium and phosphate, as well as with homeostasis [10, 11]. Compelling evidence has revealed that vitamin D deficiency is an important risk of COPD [12]. Vitamin D deficiency in serum is positively associated with pulmonary function indices in COPD patients [13–15]. However, the etiopathogenesis of vitamin D deficiency aggravating the occurrence and progression of COPD is not fully understood.

Iron is an essential mineral that maintains normal cellular function and homeostasis [16, 17], and its storage is precisely regulated by ferritin [16–18]. Ferritin can be synthesized by pulmonary epithelial cells. Intracellular iron overload in lung tissues is strongly correlated with the risk of many pulmonary diseases [19]. Glutathione peroxidase 4 (GPX4), which degrades small molecule peroxides and some lipid peroxides from various membranes, can reduce oxidative stress and lipid peroxidation [20]. The transcription factor, nuclear factor (erythroid-derived 2)-like 2 (Nrf-2) is a defense system aimed to retain cellular homeostasis [21]. Nrf-2 is an important transcription factor which activates the transcription of defense genes and plays its antioxidant role [22]. GPX4 is one of the critical antioxidant genes regulated by Nrf-2 [23]. In addition, Nrf-2 also can directly regulate several genes involved in iron metabolism, including ferritin [24]. Several laboratory experiments have revealed that cigarette smoke-induced GPX4 depression and ferritin elevation in pulmonary epithelial cells are involved in the pathophysiological mechanism of COPD [25–27]. Epidemiological studies have shown that iron and ferritin concentrations are elevated and pulmonary GPX4 expression is reduced in patients with COPD [28–30].

The previous study has revealed that vitamin D deficiency is strongly associated with the reduction of VDR and Nrf-2 in the lungs of COPD patients [15]. Moreover, vitamin D supplementation activates VDR and Nrf-2 antioxidant axis [31]. VDR inactivation induces iron parameters upregulation and lipid peroxidation in the kidney [32]. Therefore, we speculated that vitamin D deficiency aggravated COPD progression via elevating iron parameters and oxidative stress in the lungs. Consequently, the purpose of this study was to explore the associations of vitamin D with GPX4 and iron parameters in COPD patients through a case–control study.

## 2. Materials and Methods

### 2.1. Data Sources and Study Population

This case–control study was conducted in the Department of Respiratory and Critical Care Medicine, Second Affiliated Hospital of Anhui Medical University. All COPD patients were selected from the established Anhui COPD cohort (AHCC) which has been described previously [33]. COPD was diagnosed judging by the COPD (GOLD) criteria [9]: (1) the ratio of forced expiratory volume in 1 second (FEV1) to forced vital capacity (FVC) is less than 70% and FEV1% is below 80% and (2) history of smoking is more than 10 years. All enrolled COPD patients were without any treatment or intervention before hospitalization. Then, blood specimens were obtained by professional nurses.

To compare the levels of vitamin D, iron, and ferritin in serum, 172 newly diagnosed COPD patients and 172 paired with age-, gender-, and season-matched healthy volunteers were enrolled. All 172 healthy volunteers were without lung cancers, asthma, pneumonia, and other chronic pulmonary diseases and enrolled from the hospital's physical examination center. In addition, for the sake of measuring the expressions of GPX4, Nrf-2, VDR, and ferritin, lung specimens were collected from COPD patients with pneumothorax and the control group during surgery. Paratumor tissues from lung cancer patients without other chronic pulmonary diseases were regarded as the control group [34]. The demographic characteristics and clinical information are represented in Supporting Table 1. Demographic characteristics, basic diseases, and clinical information including white blood cell (WBC), neutrophil, lymphocyte, eosinophil, monocyte, basophil, C-reactive protein (CRP), and interleukin 6 (IL-6) were collected from the electronic medical record system in the hospital. The common comorbidities primarily consisted of hypertension, diabetes mellitus, coronary disease, and cerebrovascular diseases. Moreover, a pulmonary function test was performed in all COPD patients to evaluate their severity. The levels of vitamin D and iron parameters were determined in serum, and the GPX4 protein expression was evaluated in COPD patients and healthy volunteers. The counts of VDR- and Nrf-2-positive nuclei were measured in both groups. To further estimate the potential mechanism of why low vitamin D concentration was associated with the levels of iron parameters and GPX4 in COPD patients, BEAS-2B cells were incubated with 1,25(OH)_2_D_3_, the active form of vitamin D3, for 48 h, and Nrf-2 and VDR signaling were measured in human pulmonary epithelial cells.

This study was performed in accordance with the Declaration of Helsinki and its amendments. This research was approved by the Ethics Committee of the Second Affiliated Hospital of Anhui Medical University (YX2021-146). Approval and informed consent were obtained from each patient and healthy volunteer.

### 2.2. Grade of COPD

Pulmonary function was tested by spirometry by clinicians. Patients with COPD were divided into different groups based on FEV1% as follows: GOLD 1, mild patients, FEV1%≥80%; GOLD 2, moderate patients, 50%≤FEV1% < 80%; GOLD 3, severe patients, 30%≤FEV1% < 50%; and GOLD 4, very severe patients, FEV1% < 30% [35, 36].

### 2.3. Assessment of Severity

The modified Medical Research Council (mMRC) is used to evaluate dyspnea in daily living, grading from 0 (*dyspnea is felt only during strenuous activity*) to 4 (*inability to leave the house because of severe dyspnea or breathless when dressing*) [37]. mMRC score is negatively correlated with pulmonary function indices in COPD patients. The COPD assessment test (CAT) score mainly reflects the healthy condition and the effect of COPD on daily quality of life. The evaluation contents of CAT scores primarily contain cough, expectoration, chest tightness, wheezing when climbing a hill or a floor, ability to do housework, confidence when going out, sleep quality, energy, and other eight aspects. The total scores range from 0 to 40, ≤ 10 scores: mild impact; 11–20 scores: moderate impact; 21–30 scores: severe impact; and 31–40 scores: extremely severe impact [38]. The Clinical COPD Questionnaire (CCQ) consists of 10 items and is composed of three parts, including symptoms, mental state, and functional state. Patients are instructed to recall their experiences during the last week. Thus, the overall CCQ scores vary between 0 (*very good control)* and 6 (*extremely poor control*) [39]. Moreover, imaging characteristics of the lung in COPD patients and healthy volunteers were determined using computed tomography (CT).

### 2.4. Detection of Iron Parameters

All fasting blood samples were collected in the morning, and blood samples were centrifuged at 3500 rpm and stored at −80°C refrigerator in accordance with our previous research [40]. Then, serum samples were collected. Serum ferritin was detected through enzyme-linked immunosorbent assay (ELISA). Ferritin (CSB-E05187h) ELISA kits were purchased from Cusabio, Wuhan, China (https://www.cusabio.com/). All ELISA procedure was mildly amended according to the previous studies [41, 42]. The concentration of serum iron was detected using 2,4,6-tri-(2-pyridyl)-1,3,5-triazine (TPTZ) through the Beckman Coulter automatic biochemical analyzer in the clinical laboratory of the hospital [43]. According to the existing study, there were no defined normal reference values for ferritin and iron in the serum of healthy populations. According to the previous study, the upper value of the 90% reference interval was defined as the cut-off value for normal values [44]. So, the concentrations less than the upper limit of ferritin (246.0 μg/L) and iron (16.9 μg/L) were regarded as normal values, and the normal concentrations range of ferritin and iron in serum referred to the previous studies [45].

### 2.5. Cell Culture and Treatment

BEAS-2B, human pulmonary epithelial cells, was bought from the American Type Culture Collection (ATCC, USA). BEAS-2B cells were cultured in DMEM supplemented with 7.5% fetal bovine serum in a humidified chamber with 5% CO_2_/95% air at 37°C. BEAS-2B cells were seeded in cell culture dishes. When the cell density was more than 70%, 1,25(OH)_2_D_3_ (100 nM) was added to the cells [13, 14]. After 48-h coculture, the cells were washed and harvested. Then, Nrf-2 signaling was detected using western blotting.

### 2.6. Western Blotting

The total protein was extracted from lung tissues and BEAS-2B cells with cold RIPA buffer supplemented with a 1% protease inhibitor cocktail as previously described [46]. The lysates were centrifuged at 15,000 g for 20 min after ultrasonic crushed lung tissues and cells. For nuclear protein preparation, total lysate from BEAS-2B cells was suspended in a hypotonic buffer and incubated on ice for 20 min. The suspension was vortexed and centrifuged for 30 s at 15,000 g. Then, the nuclear pellet was collected, resuspended in cytoskeleton buffer, and then incubated on ice for 15 min. After centrifugation, the nuclear pellet was washed twice and obtained [47]. Finally, the protein concentration of the supernatants was detected using the BCA Protein Assay Kit (Thermo Fisher Scientific, Waltham, Massachusetts, USA). Equal amounts of protein were separated by the SDS–PAGE gel and transferred to a polyvinylidene difluoride (PVDF) membrane. Then, the membrane was blocked with 5% skimmed milk for 1.5 h at room temperature, followed by incubation with primary antibodies, including ferritin (Abcam, #ab75973), GPX4 (Abcam, #ab25066), VDR (Cell Signaling Technology, #D2K6W), Nrf-2 (Abcam, #ab62352), lamin A/C (Bioworld, #BS6019), and *β*-actin (Cell Signaling Technology, #8H10D10) for different times. The membranes were washed with PBS three times, followed by incubation with the secondary antibody for 2 h at 30°C [48]. The blots were developed with a super signal ECL luminescence (Bio-Rad, USA) and analyzed by Image J software. *β*-Actin and lamin A/C were used as the internal controls of total protein and nuclear protein, respectively.

### 2.7. Serum 25-hydroxyvitamin D (25(OH)D) Measurement

The concentration of serum 25-hydroxyvitamin D (25(OH)D) level was measured by radioimmunoassay with ^125^I labeled 25(OH)D kits as described previously [49]. All measurement was conducted in the clinical laboratory of the hospital. The concentration of 25(OH)D under 20 ng/mL was considered as vitamin D deficiency, and the status of 25(OH)D between 20 ng/mL and 30 ng/mL was considered as vitamin D insufficient [50].

### 2.8. Immunohistochemistry (IHC)

Lung tissues were fixed using formalin, embedded with paraffin, and cut in 4 μm. Pulmonary sections were dewaxed and rehydrated. Antigen retrieval was conducted and endogenous peroxidases were quenched. Pulmonary slides were incubated with VDR and Nrf-2 primary antibodies overnight at 4°C. Then, the pulmonary slides were incubated with secondary antibodies. Lastly, the pulmonary slides were counterstained with PBS after DAB reaction and hematoxylin staining. Finally, the counts of VDR- and Nrf-2-positive nuclei were calculated [51].

### 2.9. Statistical Analyses

All statistical analyses were performed using SPSS 20.0 and GraphPad Prism software. The baseline differences of different groups of means, proportions, and medians were analyzed and compared using Student's *t*-tests, chi-square tests, and Mann–Whitney *U* tests. The correlations among serum vitamin D, iron parameters, GPX4, and pulmonary function indicators were analyzed in COPD patients by Pearson correlative analysis and linear regression analysis. The relationship between VDR and Nrf-2 in lungs was assessed through Pearson correlative analysis. All *p* values less than 0.05 were considered significant.

## 3. Results

### 3.1. Basic Data of Participators

The demographic characteristics and clinical information were evaluated between COPD patients and healthy volunteers. As presented in Table 1, a total of 172 COPD patients (75.6% male) aged 43–93 years (mean 73.72 years) were recruited. No differences in age, gender, and enrolled seasons were found between COPD patients and healthy volunteers. In COPD patients, there were 126 (73.3%) former smokers and 46 (26.7) current smokers. There was a difference in smoking status between COPD patients and the control group. The numbers of WBC, neutrophils, and basophils were increased, and the count of lymphocytes was reduced in COPD patients (Table 1). The numbers of hypertension, diabetes mellitus, coronary disease, and cerebrovascular diseases were elevated in COPD patients (Table 1). In COPD patients, the levels of CRP and IL-6 were higher than those in control subjects (Table 1). 25(OH)D content was reduced, and the levels of ferritin and iron were increased in the serum of COPD patients (Table 1). The mean score of CAT was 25.56, and the median scores of mMRC and CCQ were 2.0 and 25.0, respectively (Table 1). Fifteen lung specimens were obtained from lung cancer patients (CTRL group) without other chronic diseases and COPD patients with emphysema. No differences in demographic characteristics and clinical information were found between COPD patients and control cases. As exhibited in Supporting Figure 1A, emphysema and the thicker bronchial wall were found in COPD patients. In addition, the area of centrilobular emphysema was gradually elevated in parallel with the increased grades of COPD (Supporting Figure 1B).

### 3.2. The levels of Vitamin D, GPX4, and Iron Parameters

**Figure 1:**
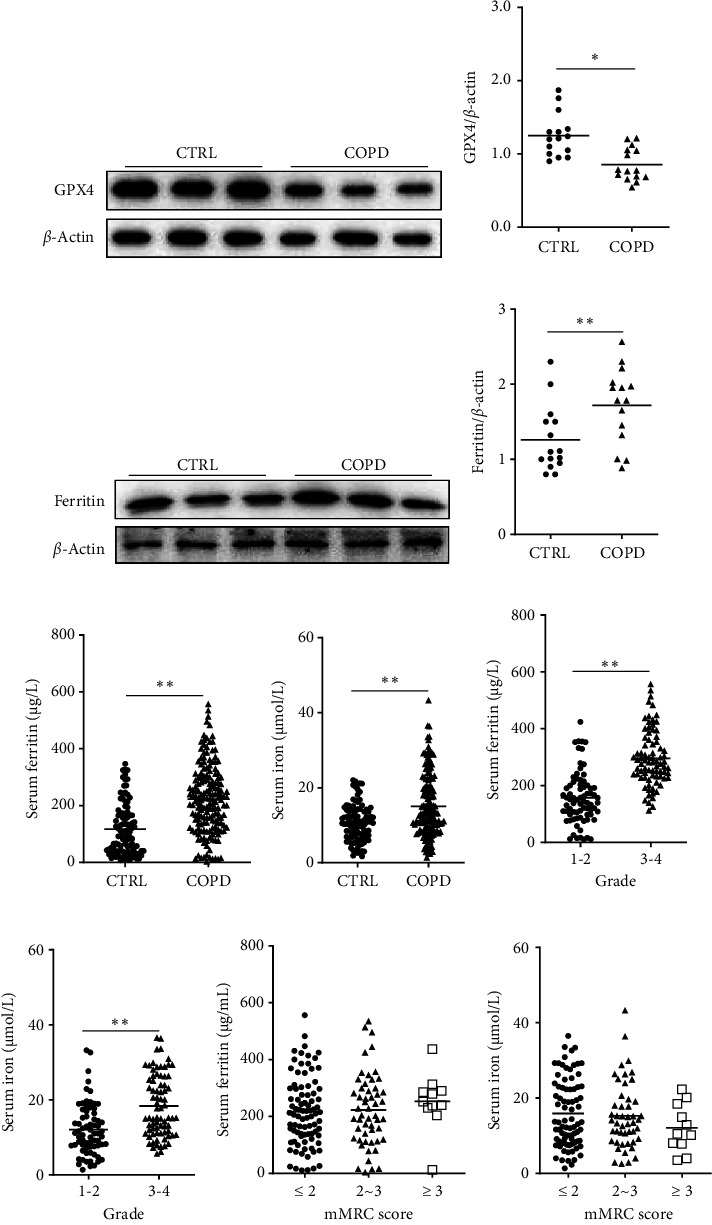
The levels of GPX4 and iron parameters in COPD patients and control cases. The expressions of pulmonary GPX4 and pulmonary and serum iron parameters were detected between COPD patients and the CTRL group. (a, b) The expression of GPX4 was detected in the lungs through western blotting between COPD patients and control cases. (a) Representative bands of GPX4. (b) Quantitative analysis of GPX4 was performed. (c, d) The expression of ferritin was measured in the lungs via western blotting between COPD patients and control cases. (c) Representative bands of ferritin. (d) Quantitative analysis of ferritin was conducted. (e, f) The levels of iron parameters were measured between COPD patients and control cases. (e) The level of serum ferritin was detected using ELISA. (f) The level of serum iron was evaluated by 2,4,6-tri-(2-pyridyl)-1,3,5-triazine (TPTZ). (g, h) The concentrations of ferritin and iron were compared in COPD patients with different severity. (g) Serum ferritin was compared in COPD patients with different grades. (h) Serum iron was evaluated in COPD patients with different grades. (i) Serum ferritin was analyzed in COPD patients with different mMRC scores. (j) Serum iron was estimated in COPD patients with different mMRC scores. ⁣^∗^*p* < 0.05. ⁣^∗∗^*p* < 0.01. COPD, chronic obstructive pulmonary disease; ELISA, enzyme-linked immunosorbent assay; TPTZ, 2,4,6-tri-(2-pyridyl)-1,3,5-triazine.

**Figure 2:**
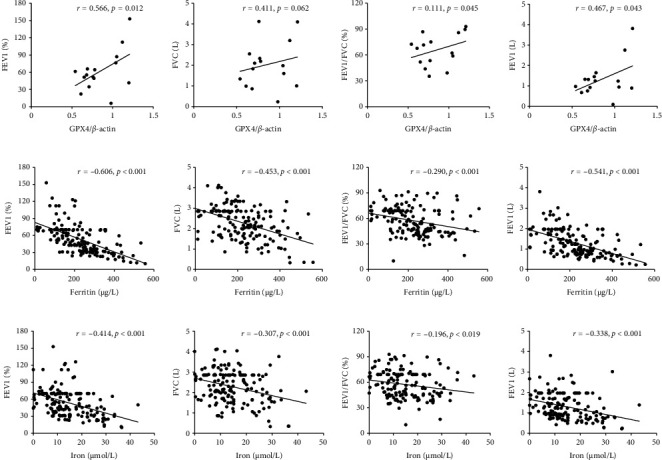
The correlations between pulmonary function with the expressions of GPX4 and iron parameters in COPD patients. (a–d) The correlations between GPX4 expression and pulmonary function indices were evaluated in COPD patients. (a) GPX4 versus FEV1%. (b) GPX4 versus FVC. (c) GPX4 versus FEV1/FVC%. (d) GPX4 versus FEV1. (e–h) The correlations between serum ferritin and pulmonary function indices were evaluated in COPD patients. (e) Serum ferritin versus FEV1%. (f) Serum ferritin versus FVC. (g) Serum ferritin versus FEV1/FVC%. (h) Serum ferritin versus FEV1. (i–l) The correlations between serum iron and pulmonary function parameters were evaluated in COPD patients. (i) Serum iron versus FEV1%. (j) Serum iron versus FVC. (k) Serum iron versus FEV1/FVC%. (l) Serum iron versus FEV1. COPD, chronic obstructive pulmonary disease; FEV1, forced expiratory volume in 1 second; FVC, forced vital capacity.

As presented in Supporting Figure 2A, serum vitamin D concentration was reduced in COPD patients compared with control subjects. Moreover, the concentration of serum vitamin D was decreased in Grade (G) 3–4 COPD patients than those in G 1-2 (Supporting Figure 2B). Meanwhile, there was no difference in serum vitamin D in COPD patients with different mMRC scores (Supporting Figure 2C). As presented in Figures 1(a) and 1(b), the expression of pulmonary GPX4 was downregulated, and the expression of pulmonary ferritin was increased in COPD patients compared with controls (Figures 1(c) and 1(d)). As presented in Figures 2(e) and 2(f), the levels of ferritin and iron were also elevated in the serum of COPD patients. In addition, the levels of ferritin and iron in serum were elevated in G 3–4 COPD patients than those in G 1-2 cases (Figures 1(g) and 1(h)). Moreover, no differences in serum ferritin and iron were observed among COPD patients with different mMRC scores (Figures 1(i) and 1(j)).

### 3.3. Associations Among GPX4, Iron Parameters, and Pulmonary Function Indicators in COPD Patients

As shown in Figures 2(a)–2(d), although there was no correlation between pulmonary GPX4 and FVC, pulmonary GPX4 expression was positively correlated with FEV1%, FEV1/FVC%, and FEV1. As presented in Figures 2(e), 2(f), and 2(h), serum ferritin and iron were negatively associated with FEV1%, FVC, FEV1/FVC%, and FEV1 (Figures 2(i), 2(j), and 2(l)).

### 3.4. Associations Among Vitamin D, Iron Parameters, and GPX4 in COPD Patients

**Figure 3:**
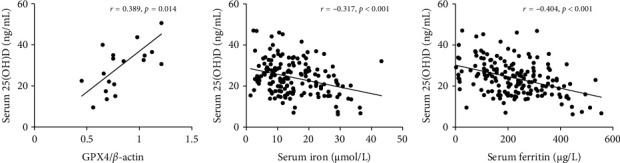
The correlations between serum vitamin D with iron parameters and GPX4 in COPD patients. (a–c) The correlations between 25(OH)D with iron parameters and GPX4 were assessed in COPD patients. (a) Pulmonary GPX4 versus serum 25(OH)D. (b) Serum iron versus serum 25(OH)D. (c) Serum ferritin versus serum 25(OH)D. COPD, chronic obstructive pulmonary disease.

As shown in Figures 3(a), 3(b), and 3(c), serum 25(OH)D concentration was positively correlated with pulmonary GPX4 and negatively associated with iron and ferritin in COPD patients. As presented in univariable linear regression, it was found that serum vitamin D reduction was associated with iron parameters' elevation. Multivariable linear regression revealed that 1-unit elevation of serum vitamin D was associated with 0.936 (95% CI: 0.894 and 0.981) μg/L and 0.941 (95% CI: 0.898 and 0.986) μmol/L declines in ferritin and iron, respectively (Supporting Table 2).

### 3.5. Associations Among VDR, GPX4, and Iron Parameters in COPD Patients

**Figure 4:**
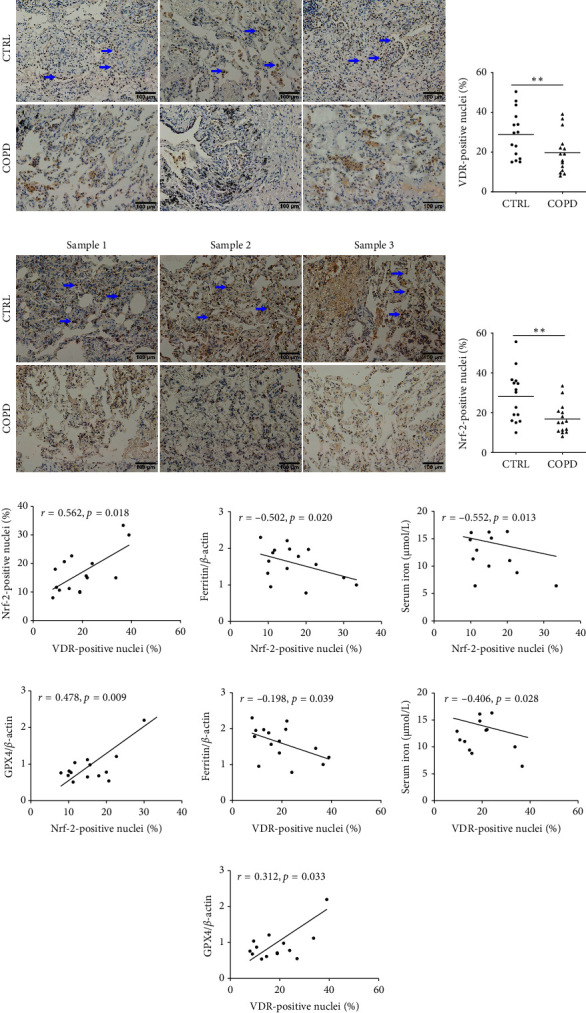
The association between VDR- and Nrf-2-positive nuclei in COPD patients. (a, b) Pulmonary VDR-positive nuclei were detected through IHC in COPD patients and control subjects. (a) Representative pictures of pulmonary VDR-positive nuclei. (b) Quantitative analysis of VDR-positive nuclei was conducted. (c, d) Pulmonary Nrf-2-positive nuclei were detected via IHC in COPD patients and control subjects. (c) Representative pictures of pulmonary Nrf-2-positive nuclei. (d) Quantitative analysis of Nrf-2-positive nuclei was conducted. (e) The correlation between VDR-positive nuclei and Nrf-2-positive nuclei was evaluated in COPD patients. (f–h) The correlations between Nrf-2-positive nuclei with iron parameters and GPX4 expression in COPD patients. (f) Nrf-2-positive nuclei versus ferritin expression. (g) Nrf-2-positive nuclei versus serum iron. (h) Nrf-2-positive nuclei versus GPX4 expression. (i–k) The correlations between Nrf-2-positive nuclei with iron parameters and GPX4 expression in COPD patients. (i) VDR-positive nuclei versus ferritin expression. (j) VDR-positive nuclei versus serum iron. (k) VDR-positive nuclei versus GPX4 expression. ⁣^∗∗^*p* < 0.01. COPD, chronic obstructive pulmonary disease; VDR, vitamin D receptor.

As illustrated in Figures 4(a), 4(b), 4(c), and 4(d), VDR- and Nrf-2-positive nuclei were reduced in pulmonary epithelial cells of COPD patients than those in control subjects. The number of VDR-positive nuclei was positively associated with Nrf-2-positive nuclei (Figure 4(e)). In addition, the number of Nrf-2-positive nuclei was negatively correlated with pulmonary ferritin and serum iron (Figures 4(f) and 4(g)). There was a positive relationship between Nrf-2-positive nuclei and GPX4 expression (Figure 4(h)). Moreover, the count of VDR-positive nuclei was inversely linked to ferritin and serum iron and positively related to GPX4 expression (Figures 4(i), 4(j), and 4(k)).

### 3.6. Vitamin D Activates Nrf-2 Signaling in Human Pulmonary Epithelial Cells

**Figure 5:**
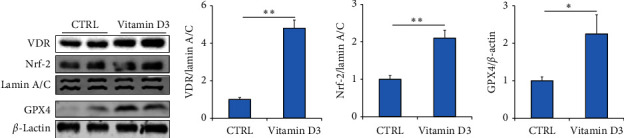
The effect of vitamin D incubation on Nrf-2 signaling in BEAS-2B cells. BEAS-2B cells were cocultured with 1,25(OH)_2_D_3_ (100 nM). After 48-h incubation, the cells were washed and harvested. Nuclear protein and total protein were extracted in BEAS-2B cells. The expressions of Nrf-2 signaling were detected using western blotting. (a) Representative bands of VDR, Nrf-2, and GPX4. (b) Quantitative analysis of VDR was conducted. (c) Quantitative analysis of Nrf-2 was conducted. (d) Quantitative analysis of GPX4 was performed. ⁣^∗^*p* < 0.05. ⁣^∗∗^*p* < 0.01. BEAS-2B, Bronchial Epithelium transformed with Ad12-SV40 2B; VDR, vitamin D receptor.

As shown in Figures 5(a), 5(b), and 5(c), 1,25(OH)_2_D_3_ pretreatment activated VDR and Nrf-2 signaling in BEAS-2B cells. Moreover, 1,25(OH)_2_D_3_ incubation significantly elevated the expression of GPX4 in BEAS-2B cells (Figure 5(d)).

## 4. Discussion

The purpose of this study was to analyze the associations among vitamin D, the core markers of iron parameters, GPX4, and pulmonary function indicators in COPD patients. The current research mostly found that (1) serum vitamin D was gradually reduced in parallel with the elevated severity in COPD patients; (2) the levels of iron parameters were increased and the expression of pulmonary GPX4 was decreased in COPD patients; (3) pulmonary function was inversely associated with iron parameters and positively correlated with GPX4 in COPD patients; (4) serum vitamin D concentration was positively associated with pulmonary GPX4 expression and negatively associated with iron parameters levels in COPD patients; (5) Nrf-2-positive nuclei was positively associated with GPX4 expression and inversely associated with iron parameters; and (6) vitamin D3 incubation activated Nrf-2 signaling in pulmonary epithelial cells.

Vitamin D is one of the important steroid hormones in humans. Over the years, more and more research studies have indicated that vitamin D exerts a significant role in maintaining the health of the respiratory system. Vitamin D deficiency has become a vital risk factor for COPD [12]. Moreover, several studies from our team have found that the content of serum vitamin D is decreased in COPD patients, and the concentration of serum vitamin D is positively associated with pulmonary function parameters among COPD patients [13, 14]. Thus, the levels of serum vitamin D were again verified in another COPD group. Those results also affirmed that serum vitamin D was reduced in COPD patients compared to healthy volunteers. In addition, serum vitamin D was gradually reduced in line with pulmonary function decline in COPD patients. This study again furnished epidemiological evidence that the concentration of vitamin D is positively related to pulmonary function in COPD patients.

Iron is obverted in both unbound and protein-bound forms in the lung tissues [19]. Ferritin, a complex of 24 subunits, consisting of two basic subunits: a heavy subunit (FTH1) and a light subunit (FTL), stores ferric (Fe^3+^) iron atoms in a soluble, nontoxic form. Therefore, iron storage is precisely regulated by ferritin in human bodies. Iron storage is very important and iron overload may cause severe oxidative stress in cells [20]. As we all know, GPX4, an antioxidant enzyme and sensor of oxidative stress, can remove excess lipid peroxides, scavenge ROS, and decrease oxidative stress [25]. The previous studies have revealed that the levels of iron and iron-related proteins including ferritin are elevated in the lungs of COPD patients [26–28], and the expression of pulmonary GPX4 is declined in COPD cases [52]. However, the correlations among serum vitamin D, iron parameters, and GPX4 were unclear in COPD patients. This case–control study has confirmed that the levels of iron and ferritin were increased in COPD patients. The expression of GPX4 was decreased in the lungs of COPD patients. Correlative analyses indicated that the expression of pulmonary GPX4 was positively associated with pulmonary function indices. On the contrary, the levels of iron and ferritin were inversely associated with pulmonary function parameters. Further analyses indicated that serum vitamin D concentration was positively associated with GPX4 expression and negatively related to the levels of iron and ferritin in COPD patients. As a result, these data demonstrated that low vitamin D concentration is positively related to GPX4 reduction and iron parameters elevation in COPD patients.

Transcription of FTL and FTH under oxidative stress is associated with Nrf-2, which regulates antioxidant response elements downstream of the individual genes [53]. Nrf-2 is a recognizable transcription factor and exerts a central function in antioxidation. It is widely known that the light and heavy chains (FTL/FTH1) and ferritin, the downstream target genes of Nrf-2, are directly regulated by Nrf-2 in the body [24, 54]. Moreover, Nrf-2 can directly or indirectly regulate GPX4 expression and function, which is responsible for GSH synthesis and involved in the process of lipid peroxidation [55, 56]. In addition, VDR is one of the nuclear receptors, and vitamin D incubation can activate VDR, which then directly binds to the DNA promoter region and regulates downstream target genes. Previous research studies have proved evidence that coculture with vitamin D can activate VDR and elevate the expression of Nrf-2 in different cell types [57–59]. Nrf-2 is a direct downstream target gene of VDR [55]. Moreover, GPX4 is also transcriptionally regulated by transcriptional factor VDR. VDR can directly bind to GPX4 promoter [32]. Ferritin presents a paradox. It not only regulates iron storage, ferritin is considered benign, but also reflects oxidative stress and arises from damaged cells [60, 61]. Oxidative stress and inflammation can evoke the elevations of iron parameters. Serum ferritin has become a vital inflammatory disease marker [62]. Hence, we speculated that low vitamin D concentration evoked GPX4 decline and iron parameters increase through repressing Nrf-2 transcriptional activity and incurring oxidative stress in pulmonary epithelial cells. Our results found that Nrf-2- and VDR-positive nuclei were reduced in the lung tissues of COPD patients. There was a positive association between VDR- and Nrf-2-positive nuclei. Moreover, Nrf-2-positive nuclei were negatively associated with iron parameters and positively associated with GPX4 expression in COPD subjects. In addition, the number of VDR-positive nuclei was inversely correlated with iron parameters and positively correlated with GPX4 expression. *In vitro*, vitamin D pretreatment promoted Nrf-2 and VDR nuclear translocation in BEAS-2B cells. In addition, vitamin D supplementation elevated the expression of GPX4 in BEAS-2B cells. Thus, these results hinted that low vitamin D concentration may evoke intracellular iron overload and oxidative stress through VDR-mediated Nrf-2 downregulation.

The current research found that vitamin D deficiency was positively associated with GPX4 reduction and iron parameters elevation among COPD patients. In addition, the level of pulmonary function gradually declined in line with serum 25(OH)D concentration decrease. Previous studies have revealed that vitamin D deficiency aggravates cigarette smoke-induced initiation and development of COPD in mice [63]. On the contrary, low-dose vitamin D3 administration can alleviate lung injury evoked by cigarette smoke in mice [64]. *In vitro* experiments from our research and another team also validated that 1,25(OH)_2_D_3_ pretreatment directly elevates Nrf-2 transcriptional activity and GPX4 expression through activating VDR [65, 66]. Therefore, these results hinted that vitamin D deficiency aggravated the progression of COPD through elevated iron parameters and incurring oxidative stress. Vitamin D supplementation may alleviate cigarette smoke-evoked COPD progression via activating VDR-mediated Nrf-2 signaling. Understanding the relationship between serum vitamin D and COPD progression may benefit disease severity and prognosis among COPD patients. Further investigations would be conducted to better explore the role of vitamin D in COPD patients.

There were several limitations in the current research. First, this was an epidemiological research, and the causal relationships between vitamin D deficiency with GPX4 reduction and iron parameters elevation were unknown. Second, this was a single-center study with a small sample size, and a larger sample from multicenter research will help to confirm these results in the future. Third, whether vitamin D deficiency associated with pulmonary intracellular iron overload and lipid peroxidation implicated in the progress of COPD was obscure. More animal experiments are needed to ascertain the associations. Fourth, the mechanism of vitamin D deficiency-evoked GPX4 decrease and ferritin increase was obscure in the current epidemiological study. Only *in vitro* and *in vivo* experiments can resolve this puzzle.

## 5. Conclusion

In summary, this investigation suggests that vitamin D concentration is inversely related to iron parameters and positively correlated with GPX4 in COPD patients. VDR-positive nuclei are positively associated with Nrf-2 and GPX4, and negatively linked to iron parameters among COPD subjects. Vitamin D supplementation activates Nrf-2 signaling and elevates GPX4 in pulmonary epithelial cells. Consequently, vitamin D deficiency may aggravate COPD progression through evoking oxidative stress and damage to the lung epithelium. Thus, vitamin D may be a potential therapeutic target for COPD patients.

## Figures and Tables

**Table 1:** Demographic information and clinical characteristics.

Variables	CTRL (*n* = 172)	COPD (*n* = 172)	*p*
Age (years)	75.41 ± 1.81	73.72 ± 0.69	0.235
Male, *n* (%)	124 (72.1)	130 (75.6)	0.100
Smoking status, *n* (%)			< 0.01
Never smoker	30 (17.4)	0	
Former smoker	67 (39.0)	126 (73.3.)	
Current smoker	75 (43.6)	46 (26.7)	
WBC (10^9^/L)	6.24 ± 0.15	7.59 ± 0.36	< 0.01
Neutrophil (10^9^/L)	3.36 ± 0.12	5.53 ± 0.35	< 0.01
Lymphocyte (10^9^/L)	2.27 ± 0.06	1.23 ± 0.07	< 0.01
Eosinophil (10^9^/L)	0.16 ± 0.01	0.19 ± 0.04	0.421
Monocyte (10^9^/L)	0.46 ± 0.02	0.62 ± 0.04	0.051
Basophil (10^9^/L)	0.02 ± 0.01	0.04 ± 0.01	0.046
Hypertension, *n* (%)	18 (10.5)	60 (34.9)	< 0.01
Diabetes mellitus, *n* (%)	8 (4.65)	19 (11.0)	0.043
Coronary disease, *n* (%)	10 (5.81)	23 (13.4)	0.027
Cerebrovascular diseases, *n* (%)	7 (4.07)	18 (10.5)	0.036
CRP (mg/L)	10.11 ± 1.35	52.72 ± 3.78	< 0.01
IL-6 (pg/mL)	30.65 ± 7.65	76.31 ± 6.20	< 0.01
25(OH)D (ng/mL)	31.32 ± 4.65	18.98 ± 5.63	< 0.01
Ferritin (μg/L)	93.65 ± 10.32	215.65 ± 11.82	< 0.01
Iron (μmol/L)	8.96 ± 1.11	14.65 ± 0.95	< 0.01
CAT	N.A.	25.56 ± 0.581	N.A.
mMRC	N.A.	2.0 (2.0, 3.0)	N.A.
CCQ	N.A.	25.0 (3.0, 34.5)	N.A.
FEV1 (L)	N.A.	1.29 ± 0.05	N.A.
FVC (L)	N.A.	2.26 ± 0.07	N.A.
FEV1 (%)	N.A.	53.03 ± 2.02	N.A.
FEV1/FVC (%)	N.A.	57.08 ± 1.32	N.A.

Abbreviation: N.A., not available.

## Data Availability

The original contributions presented in the study are included in the article. Further inquiries can be directed to the corresponding author.

## Nomenclature

CAT: COPD assessment test
CCQ: Clinical COPD Questionnaire
COPD: Chronic obstructive pulmonary disease
FEV1: Forced expiratory volume in 1 second
FVC: Forced vital capacity
GSH: Glutathione
GPX4: Glutathione peroxidase 4
mMRC: Modified Medical Research Council Dyspnea Scale
Nrf-2: Nuclear factor E2-related factor 2
VDR: Vitamin D receptor.

## Supporting Information

Supporting Figure 1. The levels of serum vitamin D in COPD patients and control cases. Serum 25(OH)D was detected through radioimmunoassay. (a) The levels of serum 25(OH)D between control cases and COPD patients. (b) The levels of serum 25(OH)D in COPD patients with different grades. (c) The levels of serum 25(OH)D in COPD patients with different mMRC scores. ⁣^∗∗^*p* < 0.01. Supporting Figure 2. Pulmonary imaging features of COPD patients and healthy volunteers. The pulmonary imaging features were evaluated through a CT scan. (a) CT images were shown between COPD patients and healthy volunteers. (b) CT images were exhibited among COPD patients with different severity grades. Supporting Table 1. Demographic information and clinical characteristics. Supporting Table 2. Linear regression analyses for the associations of vitamin D with iron parameters in COPD patients.
