# Correction to “Associations of Vitamin D With GPX4 and Iron Parameters in Chronic Obstructive Pulmonary Disease Patients: A Case–Control Study”

**DOI:** 10.1155/carj/9879624

**Published:** 2026-02-03

J. Fei, L. Liu, J.‐F. Li, et al., “Associations of Vitamin D With GPX4 and Iron Parameters in Chronic Obstructive Pulmonary Disease Patients: A Case–Control Study,” *Canadian Respiratory Journal* (2024): 2024 4505905, https://doi.org/10.1155/2024/4505905.

In the article titled “Associations of Vitamin D With GPX4 and Iron Parameters in Chronic Obstructive Pulmonary Disease Patients: A Case–Control Study,” there was an error in the Funding section, where the award ID “gxyp2021169” for the funding source the Talent Project of Colleges and Universities in Anhui Province is incorrect. The corrected funding section appears below:

**Funding**

This study was supported by National Natural Science Foundation of China (82100078), Scientific Research of Health Commission in Anhui Province (AHWJ2021b091, AHWJ2023A20170), Anhui Medical University Foundation (2021xkj159, 2023xkj208), Talent Project of Colleges and Universities in Anhui Province (gxyq2021169), Anhui Provincial Clinical Research Transformation Project (202204295107020056), Research Funds of Center for Big Data and Population Health of IHM (JKS2023010), Open Project of Anhui Province Key Laboratory of Respiratory Diseases (HX2022D01), and University Natural Science Research Project of Anhui Province (2023AH030117).

We apologize for this error.

