# Supplementary figures and images for: Associations of Vitamin D With GPX4 and Iron Parameters in Chronic Obstructive Pulmonary Disease Patients: A Case–Control Study
Source: Can Respir J. 2024 Oct 28;2024:4505905. doi: 10.1155/2024/4505905 (PMC11535414; doi:10.1155/2024/4505905)

## Slide 1
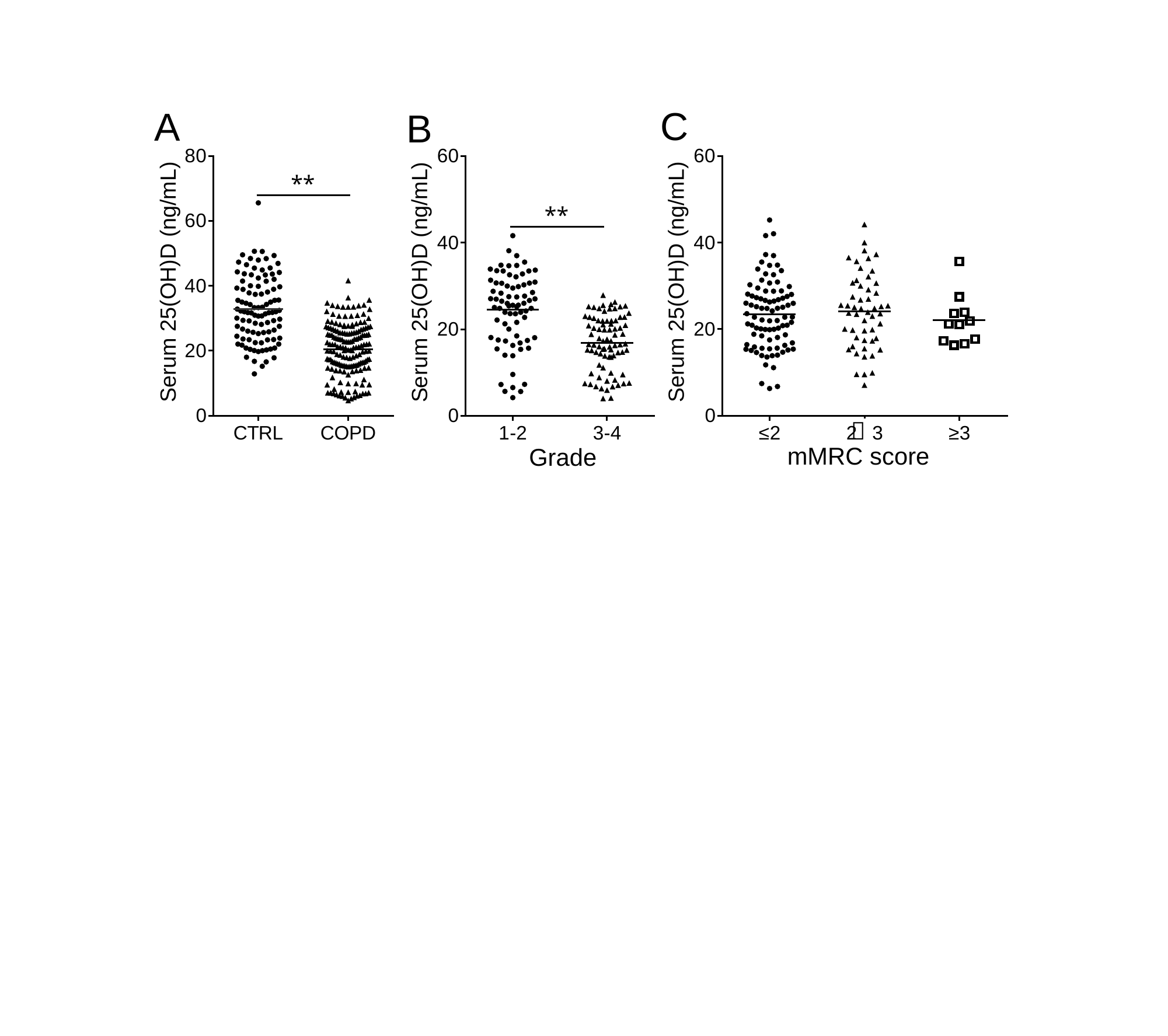

C
A
B
**
**
～
mMRC score
Grade

Supplement: Supporting Information — Additional supporting information can be found online in the Supporting Information section. [file 4505905.f1.zip › Supplemental Figure 1.pptx]

## Slide 1
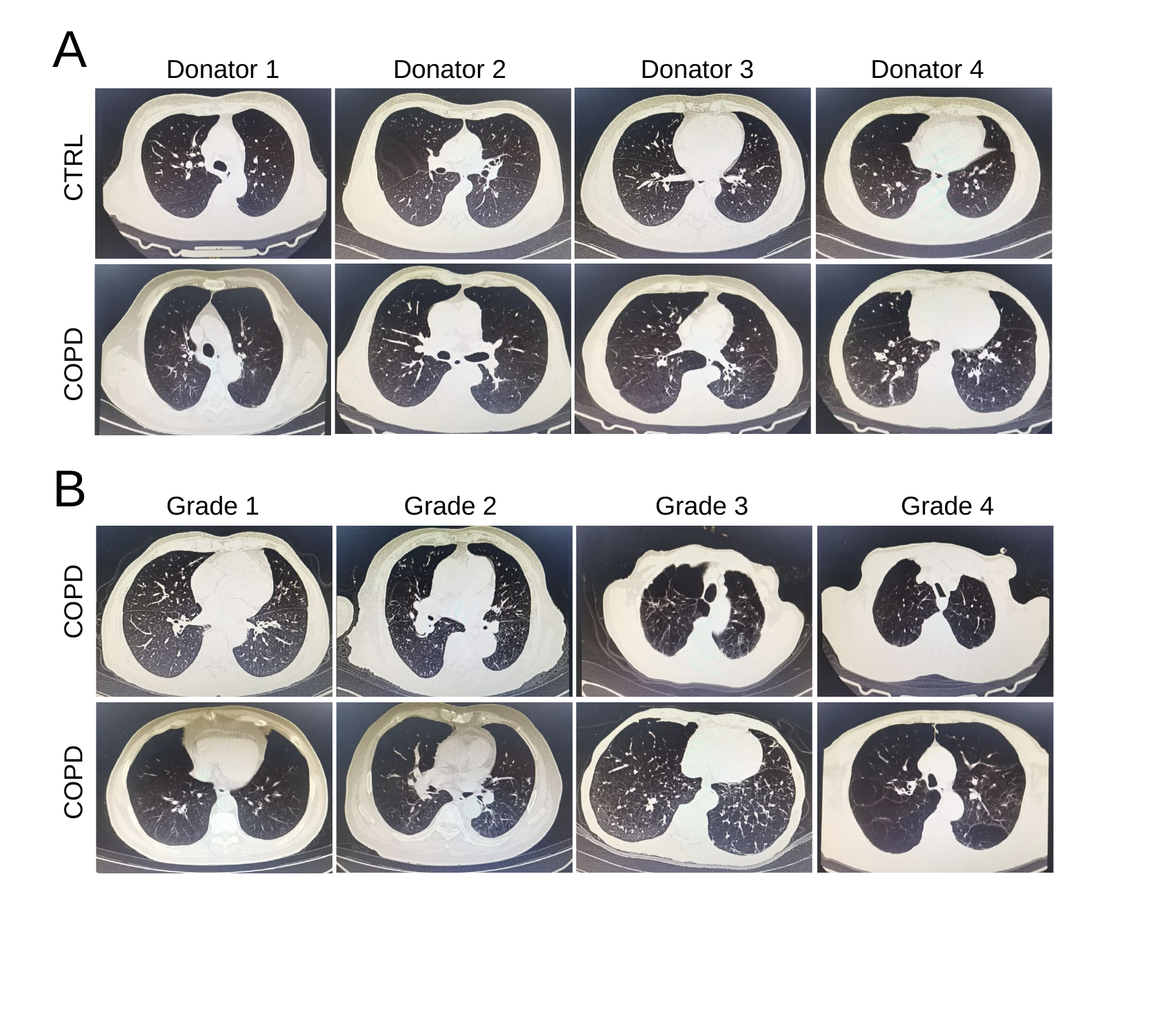

A
Donator 2
Donator 3
Donator 1
Donator 4
CTRL
COPD
B
Grade 1
Grade 2
Grade 3
Grade 4
COPD
COPD

Supplement: Supporting Information — Additional supporting information can be found online in the Supporting Information section. [file 4505905.f1.zip › Supplemental Figure 2.pptx]
