# Supplementary material for: Associations of Vitamin D With GPX4 and Iron Parameters in Chronic Obstructive Pulmonary Disease Patients: A Case–Control Study
Source: Can Respir J. 2024 Oct 28;2024:4505905. doi: 10.1155/2024/4505905 (PMC11535414; doi:10.1155/2024/4505905)
Supplement: Supporting Information — Additional supporting information can be found online in the Supporting Information section. [file 4505905.f1.zip › Supplemental Figure (1).doc]

**Supplemental Figures**

**Supplemental Figure 1. The levels of serum vitamin D in COPD patients and control cases.** Serum 25(OH)D was detected through radioimmunoassay. (A) The levels of serum 25(OH)D between control cases and COPD patients. (B) The levels of serum 25(OH)D in COPD patients with different grades. (C) The levels of serum 25(OH)D in COPD patients with different mMRC scores. ***P* < 0.01.

**Supplemental Figure 2. Pulmonary imaging features of COPD patients and healthy volunteers.** The pulmonary imaging features were evaluated through computed tomography scan. (A) CT images were shown between COPD patients and healthy volunteers. (B) CT images were exhibited among COPD patients with different severity grades.
