# Supplementary material for: Associations of Vitamin D With GPX4 and Iron Parameters in Chronic Obstructive Pulmonary Disease Patients: A Case–Control Study
Source: Can Respir J. 2024 Oct 28;2024:4505905. doi: 10.1155/2024/4505905 (PMC11535414; doi:10.1155/2024/4505905)
Supplement: Supporting Information — Additional supporting information can be found online in the Supporting Information section. [file 4505905.f1.zip › Supplemental Table 1 (2).doc]

Supplemental Table 1. Demographic information and clinical characteristics.

| Variables | CTRL (n=15) | COPD (n=15) | *P* |
| --- | --- | --- | --- |
| Age (years) | 72.78±1.52 | 72.65±1.32 | 0.326 |
| Male, n (%) | 12 (80.0) | 11 (73.3) | 1.000 |
| Smoking status, n (%) |  |  | 1.000 |
| Never-smoker | 0 | 0 |  |
| Former-smoker | 15 (10.0) | 15 (10.0) |  |
| Current-smoker | 0 (0) | 0 (0) |  |
| WBC (10^9^/L) | 7.21±0.85 | 7.42±0.65 | 0.207 |
| Neutrophil (10^9^/L) | 4.98±0.48 | 5.76±0.63 | 0.065 |
| Lymphocyte (10^9^/L) | 1.26±0.66 | 1.15±0.11 | 0.097 |
| Eosinophil (10^9^/L) | 0.33±0.09 | 0.21±0.06 | 0.118 |
| Monocyte (10^9^/L) | 0.58±0.03 | 0.74±0.06 | 0.231 |
| Basophil (10^9^/L) | 0.04±0.003 | 0.05±0.001 | 0.118 |
| Hypertension, n (%) | 5 (33.3) | 6 (40.0) | 1.000 |
| Diabetes mellitus, n (%) | 1 (6.67) | 2 (13.3) | 1.000 |
| Coronary disease, n (%) | 2 (13.3) | 3 (20.0) | 1.000 |
| Cerebrovascular diseases, n (%) | 0 | 2 (13.3) | 0.483 |
| 25(OH)D (ng/mL) | 16.32±3.08 | 19.05±4.15 | 0.068 |
| Ferritin (μg/L) | 198.35±8.57 | 210.25±9.28 | 0.125 |
| Iron (μmol/L) | 15.36±2.35 | 13.28±1.05 | 0.205 |
| CRP (mg/L) | 50.11±7.76 | 60.32±5.65 | 0.077 |
| IL-6 (pg/mL) | 44.65±5.49 | 63.28±9.21 | 0.025 |
| CAT | N.A. | 30.45±0.79 | N.A. |
| mMRC | N.A. | 2.0 (2.0, 3.0) | N.A. |
| CCQ | N.A. | 30.0 (4.0, 40.0) | N.A. |
| FEV1 (L) | N.A. | 1.11±0.08 | N.A. |
| FVC (L) | N.A. | 2.05±0.01 | N.A. |
| FEV1 (%) | N.A. | 48.62±5.68 | N.A. |
| FEV1/FVC (%) | N.A. | 50.36±4.65 | N.A. |

N.A., Not available.
