# Supplementary material for: Associations of Vitamin D With GPX4 and Iron Parameters in Chronic Obstructive Pulmonary Disease Patients: A Case–Control Study
Source: Can Respir J. 2024 Oct 28;2024:4505905. doi: 10.1155/2024/4505905 (PMC11535414; doi:10.1155/2024/4505905)
Supplement: Supporting Information — Additional supporting information can be found online in the Supporting Information section. [file 4505905.f1.zip › Supplemental Table 2 (2).doc]

Supplemental Table 2. Linear regression analyses for the associations of vitamin D with iron parameters in COPD patients.

| Variables | Univariable | *P* | Multivariable | *P* |
| --- | --- | --- | --- | --- |
|  | β (95% CI) |  | β (95% CI) |  |
| Ferritin | 0.932 (0.891, 0.974) | 0.002 | 0.936 (0.894, 0.981) | 0.006 |
| Iron | 0.950 (0.910, 0.992) | 0.019 | 0.941 (0.898, 0.986) | 0.010 |

**Note**: In the multivariable linear regression analysis, age, sex, and smoking status were adjusted.
